# Perturbations of the anti-ageing hormone Klotho in patients with type 1 diabetes and microalbuminuria

**DOI:** 10.1007/s00125-017-4219-1

**Published:** 2017-02-13

**Authors:** Giuseppe Maltese, Nikolaos Fountoulakis, Richard C. Siow, Luigi Gnudi, Janaka Karalliedde

**Affiliations:** Unit for Metabolic Medicine, Cardiovascular Division, Faculty of Life Sciences and Medicine, King’s College London, 150 Stamford Street, London, SE1 9NH UK

**Keywords:** Microalbuminuria, Soluble Klotho, Type 1 diabetes

## Abstract

**Aims/hypothesis:**

Patients with type 1 diabetes and microalbuminuria are at high risk of cardiovascular disease (CVD) and end-stage renal disease. Soluble Klotho is an anti-ageing circulating hormone involved in phosphate metabolism and vascular homeostasis through protective effects on the endothelium and antioxidant actions. The role of soluble Klotho in patients with type 1 diabetes and microalbuminuria is unknown.

**Methods:**

In a cross-sectional single-centre study we evaluated the levels of circulating serum soluble Klotho in 33 participants with type 1 diabetes and a history of microalbuminuria (receiving renin–angiotensin system [RAS] inhibitors) and 45 participants with type 1 diabetes without a history of microalbuminuria (not receiving RAS or other antihypertensive drugs). All participants had an eGFR >45 ml/min, duration of diabetes >20 years and no history of CVD. Serum soluble Klotho levels were measured by a validated immunoassay.

**Results:**

Participants with microalbuminuria had significantly lower levels of serum Klotho compared with those without microalbuminuria (median [interquartile range], 659.3 [525.3, 827.6] vs 787.7 [629.5, 1007]; *p* = 0.023). This difference persisted after adjustment for variables including age and eGFR. In a subgroup of 30 individuals with and without microalbuminuria, other markers of phosphate balance were not significantly different.

**Conclusions/interpretation:**

In individuals with type 1 diabetes, microalbuminuria is associated with soluble Klotho deficiency. Further studies are required to determine whether soluble Klotho is causally related to the development of cardio-renal disease in type 1 diabetes.

**Electronic supplementary material:**

The online version of this article (doi:10.1007/s00125-017-4219-1) contains peer-reviewed but unedited supplementary material, which is available to authorised users.

## Introduction

Microalbuminuria (MA) is one of the earliest manifestations of diabetic kidney disease (DKD) in type 1 diabetes [1]. MA, a marker of endothelial dysfunction, is associated with a higher risk of cardiovascular disease (CVD) morbidity and mortality [1]. Increased oxidative stress and endothelial dysfunction are key events contributing to the pathogenesis of DKD and CVD [1].

Klotho is a nephroprotective transmembrane protein predominantly expressed in the renal tubules and implicated in regulating phosphate metabolism, together with fibroblast growth factor-23 (FGF-23) [2]. A circulating form of Klotho, named soluble Klotho (resulting from a proteolytic cleavage of the transmembrane protein), is detectable in the circulation and has been demonstrated to maintain vascular homeostasis through antioxidant properties [2]. In vivo, soluble Klotho deficiency is accompanied by activation of the renin angiotensin system (RAS) and endothelial dysfunction [2]. Individuals with type 2 diabetes and an eGFR >60 ml/min have reduced tissue levels of Klotho compared with individuals with IgA nephropathy [3]. ESM Table 1 summarises human and in vivo studies where Klotho levels have been associated with DKD. In rodent models of type 1 diabetes, renal expression of Klotho is reduced [3]. To date there is a lack of information on the relationship between circulating soluble Klotho and MA in patients with type 1 diabetes.

## Methods

This study included 78 individuals with type 1 diabetes attending the diabetes outpatient clinic at Guy’s Hospital, London, UK. Of these, 33 had microalbuminuria (MA^+^) and 45 had normoalbuminuria (MA^−^). All study participants gave informed consent and investigations were approved by the responsible ethics committee. Clinical details and biochemical measurements for the cohort are shown in Table 1. For measurement of serum Klotho, blood samples were immediately centrifuged at 1500*g* at 4°C for 10 min and the supernatant fractions were stored at 80°C (for <24 months) with no freeze–thaw cycles before analysis. This approach has been shown not to impact on the sensitivity of the assay used in this study [4]. All samples were assayed in duplicate using an immunoassay kit (Immuno-Biological-Laboratories, Hamburg, Germany) [4–6].

**Table 1:** Comparison of the clinical and biochemical features of 78 type 1 diabetes participants with (MA^+^) and without (MA^−^) microalbuminuria

Variable	MA^+^ (*n* = 33)	MA^−^ (*n* = 45)	*p* value
Age, years	54.4 ± 11.6	43.3 ± 9.6	<0.001
Sex, % male (M/F)	70 (23/10)	44 (20/25)	0.03
Retinopathy, %	100	100	NS
Use of RAS inhibitors, %	100	0	<0.001
Use of statins, % (*n*)	70 (23)	29 (13)	<0.001
Diabetes duration, years	32.7 ± 10.2	29.5 ± 9.7	0.21
HbA_1c_, %	7.7	7.9	0.30
HbA_1c,_ mmol/mol	60.9 ± 10.7	63.0 ± 10.0	
SBP, mmHg	132.6 ± 12.4	126.3 ± 13.1	0.06
DBP, mmHg	72.3 ± 9.2	74.8 ± 8.5	0.26
eGFR, ml/min	90.2 ± 21.7	100.2 ± 20.4	0.054
ACR, mg/mmol^a^	1.1 (0.5, 8.1)	0.7 (0.5, 1.05)	0.013
Soluble Klotho, pg/ml^a^	659.3 (525.3, 827.6)	787.7 (629.5, 1007)	0.023

All data are mean ± SD unless otherwise stated

^a^Median (interquartile range)

DBP, diastolic BP; SBP, systolic BP

All participants had an eGFR >45 ml/min, duration of diabetes >20 years and no history of CVD. Participants in the MA^+^ group had a positive history of MA (more than two early morning urine albumin to creatinine ratio (ACR) measurements ≥3.0 mg/mmol), confirmed diabetic retinopathy and were receiving RAS inhibitor treatment. The participants in the MA^−^ group were not receiving any RAS inhibitor treatment or any other antihypertensive agents.

An investigator blinded to MA status performed the soluble Klotho measurements. Urine ACR was measured from morning urine samples. Urine creatinine (Jaffe reaction) and albumin concentrations were measured by immunoturbidimetry, serum total cholesterol (enzymatic colorimetry) and creatinine (rate reaction method) levels were measured using a Cobas Mira Plus analyser (Roche Diagnostics, Rotkreuz, Switzerland) [6]. HbA_1c_ was measured by boronate affinity HPLC (Primus CLC330, Kansas City, MO, USA). eGFR was determined using the Chronic Kidney Disease Epidemiology Collaboration Formula (CKD-EPI) [7].

Descriptive statistics were used for the analysis of demographic and clinical features. Participants with and without MA were compared by an unpaired *t* test (for continuous normally distributed variables), Mann–Whitney test (for continuous variables not normally distributed) and χ^2^ test (for categorical variables). Testing for normality was performed using the Shapiro–Wilk test. Since soluble Klotho levels and ACR were not normally distributed, their values were log transformed (base *e*) for Pearson correlation analysis and multivariate logistic regression analysis. Multivariate logistic regression analysis was performed to evaluate if the significant association between MA status and Klotho levels persisted when adjusted for other variables. Data are given as mean ± SD, percentage for categorical variables, or median and interquartile range for variables not normally distributed. A two-tailed *p* value <0.05 was considered significant. Statistical analyses were performed with SPSS version 19.0 (SPSS, Chicago, IL, USA).

## Results

Participants in the MA^+^ group were older (54.4 ± 11.6 vs 43.3 ± 9.6 years), more frequently male (70% vs 44%) and more frequently on statin treatment (70% vs 29%) compared with the MA^−^ group (*p* < 0.05 for all; Table 1). MA^+^ participants also had higher systolic BP (132.6 ± 12.4 vs 126.3 ± 13.1 mmHg), lower eGFR (90.2 ± 21.7 vs 100.2 ± 20.4 ml/min) and lower levels of serum Klotho (659.3 [525.3, 827.6] vs 787.7 [629.5, 1007] pg/ml) compared with MA^−^ participants (*p* < 0.05 for all; Table 1). Glycaemic control was not significantly different between the two groups. In a multivariate logistic regression analysis, serum soluble Klotho levels were inversely and significantly associated with MA^+^ independent of other variables, including age and eGFR (ESM Table 2). A significant correlation between soluble Klotho and eGFR was not observed in the entire cohort (MA^+^ and MA^−^) or MA^+^ population.

No significant association between ACR and soluble Klotho was observed. In participants with MA^+^ (all receiving RAS inhibitors) there was no significant difference in soluble Klotho levels between those with remission of MA (ACR <3) vs those with ACR ≥3.

Other variables including 25-hydroxy vitamin D (measured by liquid chromatography and electrospray ionisation tandem MS), serum corrected calcium (albumin measured by the Roche bromocresol green method and calcium using 5‑nitro‑5′‑methyl‑BAPTA method), serum phosphorus (measured using Roche molybdate method) and intact parathyroid hormone (PTH; measured by the Roche method: sandwich assay) levels were available in a subgroup of the full cohort (*n* = 30; MA^+^, *n* = 12; MA^−^, *n* = 18) (Table 2). These patients were matched for age, HbA_1c_, BP, diabetes duration and eGFR. In this subgroup, no statistically significant differences were found in 25-hydroxy vitamin D levels, corrected calcium and PTH between the MA^+^ and MA^−^ groups. Klotho remained significantly lower in participants in the MA^+^ group compared with those in the MA^−^ group. There was a significant negative correlation between serum phosphorus and serum soluble Klotho (Pearson correlation *r* = −0.397, *p* = 0.03; ESM Fig. 1).

**Table 2:** Comparison of the clinical and biochemical features, and markers of phosphate balance in a subgroup of 30 type 1 diabetes participants with (MA^+^) and without (MA^−^) microalbuminuria

Variable	MA^+^ (*n* = 12)	MA^−^ (*n* = 18)	*p* value
Age, years	55.08 ± 8.8	47.8 ± 10.7	0.06
Diabetes duration, years	34.4 ± 10.4	31.25 ± 10.44	0.4
HbA_1c_, % (mmol/mol)	7.6 (59.43 ± 6.33)	8.0 (63.76 ± 9.07)	0.32
eGFR, ml/min	77.2 ± 28.4	93.9 ± 24.0	0.09
ACR, mg/mmol^a^	2.4 (1, 8)	0.75 (0.6, 1)	0.03
Soluble Klotho, pg/ml^a^	674.2 (571.8, 915.4)	907.3 (698.7, 1026.1)	0.047
Serum phosphorus, mmol/l	1.05 ± 0.22	0.93 ± 0.13	0.12
Corrected serum calcium, mmol/l	2.36 ± 0.08	2.35 ± 0.08	0.6
25-hydroxy vitamin D, nmol/l	51.8 ± 25.2	60.0 ± 27.0	0.3
Intact PTH, ng/l	42.8 ± 31.9	33.1 ± 16.5	0.3

All data are mean ± SD unless otherwise stated

^a^Median (interquartile range)

## Discussion

This is the first study to demonstrate that individuals with type 1 diabetes and MA^+^ have significantly lower serum soluble Klotho levels compared with MA^−^ individuals.

Our findings are consistent with in vivo data showing that Klotho deficiency has a negative impact on albumin excretion and is associated with kidney hypertrophy and exaggerated expansion of the mesangial matrix in renal glomeruli [8]. A nephroprotective role has been demonstrated for Klotho in an animal model of type 1 diabetes, with global overexpression ameliorating the histological features of DKD and reducing albuminuria [9]. In a recent study using a non-diabetic animal model of albuminuria, treatment with exogenous soluble Klotho resulted in a reduction of urinary albumin excretion by protecting against podocyte injury [10].

In individuals with type 2 diabetes, the level of plasma soluble Klotho has been shown to correlate negatively with the development of albuminuria and with a decline in eGFR [11].

Soluble Klotho levels reported in healthy adults, measured with the same assay used in the present study, are similar to our findings in individuals with type 1 diabetes [5, 12].

Soluble Klotho has antiapoptotic effects on vascular endothelial cells and protects against endothelial dysfunction [2]. The association, if any, between levels of soluble Klotho and CVD or renal endpoints in type 1 or type 2 diabetes is unknown. In non-diabetic elderly individuals, higher soluble Klotho levels are independently associated with a lower risk of CVD [13].

Increased activation of the RAS characterises DKD, whereas treatment with RAS inhibitors lowers the incidence of cardio-renal events in DKD [1]. Of interest, activation of the RAS induces a reduction in serum soluble Klotho levels [2]. We have previously demonstrated in individuals with type 2 diabetes and MA^+^ that RAS inhibition increases soluble Klotho levels [6]. In the present study, despite all participants with MA^+^ receiving RAS inhibitors, this group had significantly lower levels of serum soluble Klotho.

Our work has several limitations. A causal relationship between lower soluble Klotho and development of MA cannot be inferred as this was a cross-sectional study. Our sample size is small as we specifically excluded patients with advanced renal dysfunction and CVD, which are known to influence Klotho levels [2]. We did not measure 24 h urine excretion of calcium or phosphate or FGF-23 levels, and the results of other markers of calcium phosphate metabolism were only available for around one-third of participants. However, we did not observe any differences in these markers in our study subcohort with relatively preserved eGFR. This is consistent with the general consensus that patients with lower eGFR and advanced DKD exhibit significant perturbations in markers of calcium phosphate metabolism/turnover. In the study subcohort we noted significantly lower levels of circulating Klotho in the MA^+^ group and this may suggest that changes in Klotho occur early in type 1 diabetes DKD and before overt changes in other markers of calcium and phosphorus metabolism.

The strengths of this study are that all of the enrolled patients had relatively preserved renal function (eGFR >45), a similar long duration of diabetes and were a well-characterised cohort attending a single centre for their diabetes care.

Further prospective studies will have to elucidate the contribution of soluble Klotho deficiency to the development of MA and progression of DKD. The need for such studies is underscored by the laboratory evidence that Klotho is involved in the pathogenesis of MA and DKD.

Klotho may be a potential target to reduce or significantly slow the progression of renal disease in individuals with type 1 diabetes. With this perspective, our results establish a platform to address this in future clinical studies.

## Electronic supplementary material

Below is the link to the electronic supplementary material.

ESM 1 (PDF 36.8 kb)

## Abbreviations

ACR: Albumin to creatinine ratio
CVD: Cardiovascular disease
DKD: Diabetic kidney disease
FGF-23: Fibroblast growth factor-23
MA: Microalbuminuria
MA^+^: Presence of microalbuminuria
MA^−^: Normoalbuminuria
PTH: Parathyroid hormone
RAS: Renin–angiotensin system
