# Supplementary material for: Perturbations of the anti-ageing hormone Klotho in patients with type 1 diabetes and microalbuminuria
Source: Diabetologia. 2017 Feb 13;60(5):911–4. doi: 10.1007/s00125-017-4219-1 (PMC6518370; doi:10.1007/s00125-017-4219-1)

Supplement Table 1

Summary of recent Human and *In Vivo* studies where Klotho levels have been associated with diabetic kidney disease

**Study population**	**Main findings**
147 patients with type 2 diabetes mellitus with estimated glomerular filtration rate (eGFR) >60 ml/min and 25 healthy control subjects [1]	Soluble Klotho levels were significantly higher in patients with type 2 diabetes mellitus compared to controls. Klotho levels were inversely associated with increasing degree of albuminuria
109 patients with type 2 diabetes mellitus and 32 healthy controls [2]	Soluble Klotho levels were significantly elevated in patients with type 2 diabetes mellitus compared to controls and decreased with increasing urine albumin excretion
462 patients with type 2 diabetes mellitus and preserved renal function divided into normoalbuminuric, microalbuminuric, macroalbuminuric cohorts and 160 healthy controls [3]	Compared to controls, soluble Klotho levels were significantly decreased in patients with type 2 diabetes mellitus. Soluble Klotho levels negatively correlated with albuminuria in patients with type 2 diabetes mellitus
109 patients with type 2 diabetes and eGFR >60 ml/min. Patients were followed for median 34 months [4]	After adjusting for baseline eGFR and urine ACR, soluble Klotho was significantly associated with the decline of eGFR
31 patients with diabetic nephropathy (type 2 diabetes mellitus), 31 patients with IgA nephropathy and 7 patients with minimal changes disease [5]	Lower levels of Klotho expression in renal biopsies of patients with diabetic nephropathy when compared to patients with IgA nephropathy and minimal changes disease
146 patients with type 2 diabetes mellitus and different stages of chronic kidney disease (CKD) [6]	Compared to patients without CKD, soluble Klotho levels were decreased in early stage CKD (stage 1 and 2) and increased in advanced stages of CKD (3-5) parallel to fall in eGFR.
***In vivo* studies**	**Main findings**
Akita mice (animal model of type 1 diabetes) [7]	Klotho mRNA and protein expression lower in Akita mice compared to wild type
Mice with streptozotocin-induced type 1 diabetes [8]	*In vivo* Klotho deficiency exacerbated streptozotocin-induced increase in albuminuria, expansion of mesangial matrix in renal glomeruli, and kidney hypertrophy

References:

1. Lee EY, Kim SS, Lee JS, Kim IJ, Song SH, Cha SK, et al. Soluble alpha-klotho as a novel biomarker in the early stage of nephropathy in patients with type 2 diabetes. PloS one. 2014;9(8):e102984.
2. Inci A, Sari F, Coban M, Olmaz R, Dolu S, Sarikaya M, et al. Soluble Klotho and fibroblast growth factor 23 levels in diabetic nephropathy with different stages of albuminuria. Journal of investigative medicine : the official publication of the American Federation for Clinical Research. 2016;64(6):1128-33.
3. Wu C, Wang Q, Lv C, Qin N, Lei S, Yuan Q, et al. The changes of serum sKlotho and NGAL levels and their correlation in type 2 diabetes mellitus patients with different stages of urinary albumin. Diabetes research and clinical practice. 2014;106(2):343-50.
4. Kim SS, Song SH, Kim IJ, Lee EY, Lee SM, Chung CH, et al. Decreased plasma alpha-Klotho predict progression of nephropathy with type 2 diabetic patients. Journal of diabetes and its complications. 2016;30(5):887-92.
5. Asai O, Nakatani K, Tanaka T, Sakan H, Imura A, Yoshimoto S, et al. Decreased renal alpha-Klotho expression in early diabetic nephropathy in humans and mice and its possible role in urinary calcium excretion. Kidney International. 2012;81(6):539-47.
6 . Kacso IM, Bondor CI, Kacso G. Soluble serum Klotho in diabetic nephropathy: relationship to VEGF-A. Clinical biochemistry. 2012;45(16-17):1415-20.
7. Kadoya H, Satoh M, Haruna Y, Sasaki T, Kashihara N. Klotho attenuates renal hypertrophy and glomerular injury in Ins2Akita diabetic mice. Clin Exp Nephrol. 2016;20(5):671-678.
8. Lin Y, Kuro-o M, Sun Z. Genetic deficiency of anti-aging gene klotho exacerbates early nephropathy in STZ-induced diabetes in male mice. Endocrinology 2013;154(10):3855-63.

Supplement table 2
Multivariate logistic regression models of the relationship between soluble Klotho and Microalbuminuria in patients with type 1 diabetes and relatively preserved renal function (eGFR >45 ml/min)

	Odds Ratio	95% CI	p value
Unadjusted model	0.21	0.05 to 0.87	0.032
Model adjusted for Age	0.20	0.04 to 0.93	0.040
Model adjusted for Age and eGFR	0.22	0.05 to 0.97	0.046
Model adjusted for Age, eGFR and Statin treatment	0.12	0.02 to 0.71	0.020
Model adjusted for Age, eGFR, Statin treatment and SBP	0.13	0.02 to 0.79	0.027

Abbreviations: eGFR=estimated glomerular filtration rate, SBP = systolic blood pressure

Supplement Figure 1
Significant negative correlation between serum phosphorus and serum soluble Klotho in a sub-group of 30 type 1 diabetes patients with and without microalbuminuria

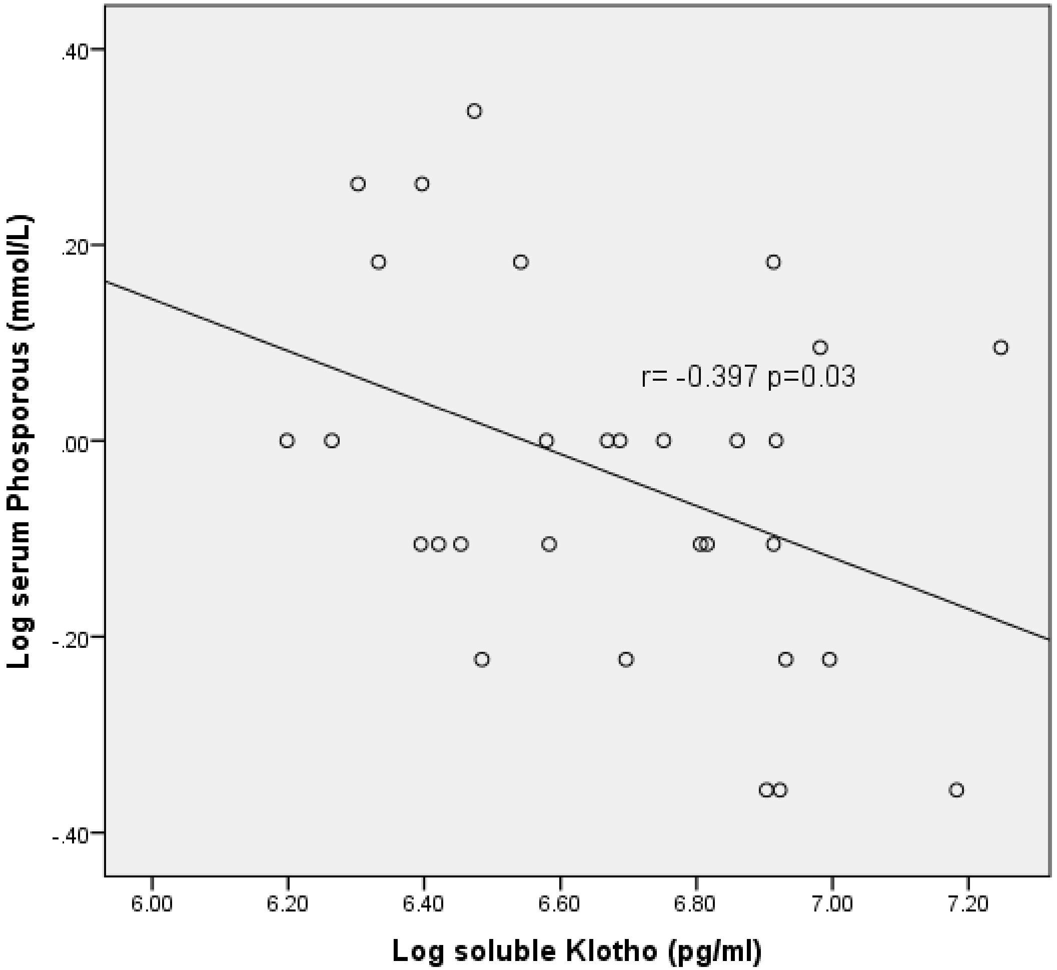

Supplement: Supplementary file 1 — (PDF 36.8 kb) [file 125_2017_4219_MOESM1_ESM.pdf]
